# DEPRESSIONAMONG PATIENTS WHO SURVIVED COVID 19 INTHE EMERGENCY DEPARTMENT

**DOI:** 10.1192/j.eurpsy.2023.1636

**Published:** 2023-07-19

**Authors:** K. Hamrouni, R. Youssef, K. Hlimi, A. Loghmari, W. Houas, H. Yaacoubi, L. Boukadida, H. Ben Salah, M. Ben othmen, I. Khalifa, R. Jaballah, A. Zorgati, R. Boukef

**Affiliations:** 1 Emergency Department; 2 Urology, Sahloul teachin Hospital Sousse, Sousse, Tunisia

## Abstract

**Introduction:**

The current SARS cov 2 virus disease pandemic presents a threatening physical danger as well as its impact on mental health. It seems important to screen any patient who has experienced covid 19 for its psychological impact.

**Objectives:**

Therefore the aim of our study was to highlight the impact of COVID-19 infection on mental health by screening them for depression

**Methods:**

This is a cross-sectional, single-center study, conducted at the Sahloul Emergency Department, over a period of 5 months (January to May 2021). Patients are collected from a database of ouremergencydepartment COVID-19 unit.We included patients whose age is greater than or equal to 18 years old; who has been infected with SARS-COV-2 according to the results of the PCR test. All patients lost to follow-up, refusing to participate in this study, having a psychiatric illness or having taken a psychotropic medication before randomization or non-cooperating (unable to respond to the evaluation test) were excluded.A telephone follow-up was done after 30 days from admission to calculate the HAMILTON score after a positif HAD D scale.

**Results:**

200 patients were included. For the 20 patients (10%) with depressive symptomatology (doubtful and certain) according to the HAD D scale, their responses to the Hamilton scale were analysed in order to determine the severity of the depressive symptomatology. Note that 30% of patients had mild to moderate signs of depression and 70% had severe depression.The majority of patients in whom the presence of depressive symptoms was noted had an average age of 40 years; those who presented with severe depression had an average age of 51.3 years with a female majority of 72%. No difference was noted in relation to pathological history, half had been hospitalized for treatment of COVID-19(57%). Patients with severe depression symptom resolution lasted an average of 14 days.

**Conclusions:**

According to the results of this study, interventions may be carried out to minimize the pandemic’s negative psychological consequences.

**Disclosure of Interest:**

None Declared

